# Theoretical studies on glycolysis of poly(ethylene terephthalate) in ionic liquids†

**DOI:** 10.1039/c7ra13173a

**Published:** 2018-02-21

**Authors:** Zhaoyang Ju, Weihua Xiao, Xingmei Lu, Xiaomin Liu, Xiaoqian Yao, Xiaochun Zhang, Suojiang Zhang

**Affiliations:** College of Engineering, China Agricultural University Beijing 100083 P. R. China; Key Laboratory of Green Process and Engineering, Beijing Key Laboratory of Ionic Liquids Clean Process, State Key Laboratory of Multiphase Complex Systems, Institute of Process Engineering, Chinese Academy of Sciences Beijing 100190 P. R. China xqyao@ipe.ac.cn sjzhang@ipe.ac.cn

## Abstract

Ionic liquids (ILs) present superior catalytic performance in the glycolysis of ethylene terephthalate (PET). To investigate the microscopic degradation mechanism of PET, density functional theory (DFT) calculations have been carried out for the interaction between ILs and dimer, which is considered to symbolize PET. We found that hydrogen bonds (H-bonds) play a critical role in the glycolysis process. In this study, 24 kinds of imidazolium-based and tertiary ammonium-based ILs were used to study the effect of different anions and cations on the interaction with PET. Natural bond orbital (NBO) analysis, atoms in molecules (AIM) and reduced density gradient (RDG) approaches were employed to make in-depth study of the nature of the interactions. It is concluded that the interaction of cations with dimer is weaker than that of anions and when the alkyl chain in the cations is replaced by an unsaturated hydrocarbon, the interaction will become stronger. Furthermore, anions play more important roles than cations in the actual interactions with dimer. When the hydrogen of methyl is replaced by hydroxyl or carboxyl, the interaction becomes weak for the amino acid anions and dimer. This work also investigates the interaction between dimer and ion pairs, with the results showing that anions play a key role in forming H-bonds, while cations mainly attack the oxygen of carbonyl and have a π-stacking interaction with dimer. The comprehensive mechanistic study will help researchers in the future to design an efficient ionic liquid catalyst and offer a better understanding of the mechanism of the degradation of PET.

## Abstract 2

Co-interaction lead to glycolysis of ethylene terephthalate (PET) in ionic liquids (ILs): H-bonds and π-stacking.

## Graphical abstract

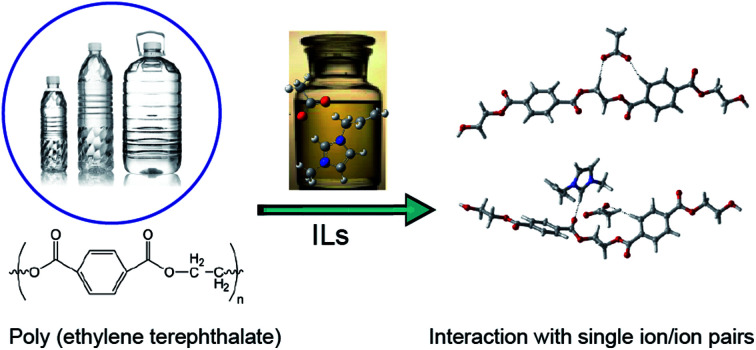

## 1. Introduction

Poly(ethylene terephthalate) (PET) is a thermoplastic polymer resin^1,2^ that is widely used in our daily lives. PET can be synthesized from fossil energy resources and biomass.^3,4^ Because of its excellent mechanical properties and chemical stability, the applications of PET are very extensive, such as in synthetic fibers, water and soft-drink bottles, food packaging, plastic films and insulation materials.^5,6^ The amount of waste plastic has increased rapidly with the development of the plastic industry. The annual world consumption of PET has exceeded 50 million tons and is still increasing markedly owing to its widespread use in the fields of bottles, packaging and fibers.^7,8^ Among different PET recycling methods, chemical recycling has been extensively investigated as the most effective method to convert PET into monomers and oligomers. There are some methods for the recycling of PET, such as methanolysis, glycolysis, hydrolysis and aminolysis.^9–11^ However, there are many unfavorable factors in these methods, including: the organic solvents (like methanol) used in the reaction process; pollution of the environment; harsh reaction conditions like high temperature and pressure; complex separation processes, *i.e.*, the raw materials, products, and acid–base catalyst medium are difficult to separate.^12–15^ Therefore, a green solvent for the depolymerization of PET needs to be developed.

As a new type of green solvents, ionic liquids (ILs) have many advantages, such as thermal stability, low vapor pressure, adjustable cations and anions, non-flammability and special solubility.^16,17^ They have attracted much attention as greener replacements for traditional volatile organic solvents in various fields, such as catalytic reactions, organic synthesis, functional materials, electrochemical reactions and life sciences.^18–24^ Kamimura *et al.* reported that ILs were first applied as solvents for 6-nylon.^25^ It led to continued and increasing explosion of interest in the recycling of PET. Originally, the ionic liquid catalysts used to study the degradation of PET included some conventional ILs, such as: BmimCl,^26^ BmimBr, BmimHSO_4_,^10^ BmimBF_4_,^27^ and AmimCl.^28^ Al-Sabagh *et al.* reported that PET could be effectively catalyzed by 1-butyl-3-methylimidazolium acetate ([Bmim]OAc) with a solubility of about 100% in EG (ethylene glycol) solvent and the selection of BHET was about 60% at 190°.^29^ Then, [Bmim]ZnCl_3_ and [Bmim]FeCl_4_ were viewed as better catalysts than [Bmim]OAc and [Bmim]Cl.^30,31^ Wang *et al.* reported the glycolysis of PET in different conditions catalyzed by tertiary ammonium-based ILs, which show good catalytic activity for the degradation of PET.^32^ In our previous work, the H-bonds formed among ILs, PET and EG were shown to play a key role in the glycolysis of PET.^32,33^ The synergic effect of all H-bonds finally results in the long chain of PET being disconnected and accelerates the degradation rate. Although these studies threw some light on the degradation mechanism, few theoretical studies have been reported on the interaction and glycolysis in the system of PET and ILs.

**Scheme 1:**
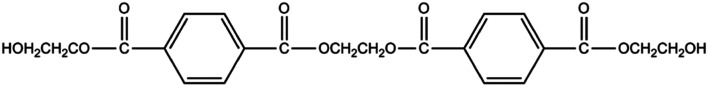
Structure of dimer.

In this work, a series of ILs including imidazolium-based cations (Amim, C_3_mim, Bmim and C_4_dmim), tertiary ammonium-based (N_1111_ and N_2222_), (OAc, Ala, Asp, Ser, HSO_4_ and Cl) anions is used to study the effects of anion and cation structures on the degradation of PET and explore the detailed interactions, especially H-bonding interactions between the PET model compound and ILs. Owing to limited computational capacity, PET is modeled by the dimer (Scheme 1), which consists of BHET units connected with C–O linkages, and this structure is thought to represent the building blocks of natural PET.^34^ We mainly study the effects of alkyl chain length, unsaturated bonds and the role of hydrogen in the C2 position in the cations as well as the effects of hydrogen's electronegativity in the amino acid anion replaced by hydroxyl or carboxyl and their coordination ability on the strength of the interaction in the anions. Interaction sites are studied to determine which positions are helpful for cleavage of the C–O linkage. Interaction energies are used to approximately evaluate which is the most efficient catalyst among the studied ILs. Natural bond orbital (NBO) analysis, atoms in molecules (AIM) theory, and reduced density gradient (RDG) are used in combination to specifically provide insight into the hydrogen bonds (H-bonds) at the molecular level.

## 2. Computational methods

All the DFT calculations are carried out with Gaussian 09 package.^35^ The geometries of dimer, EG, anions, cations and the complexes of dimer/ILs/EG are fully optimized at the B3LYP/6-31++G** level, which could be used in the studies of noncovalent interactions^36,37^ and performed with CYL view^38^ at the same level to confirm the existence of H-bonds. Likewise, some studies have achieved reasonable results in correspondence with experiments by using the same level of theory.^39–42^ Vibrational frequencies are calculated to ensure no imaginary frequencies for all the configurations. To get comparable dispersion-corrected results,^43,44^ DFT is also applied to all the geometries to determine π-stacking interactions at the B3LYP-D3/6-311+G** level of theory. The electrostatic potential (ESP) method is used to predict the most plausible site for electrophilic and nucleophilic attack.^45^ The electrostatic potential surfaces for the most stable geometries of dimer, EG, anions and cations are given in Fig. S1.† The interaction energy (Δ*E*) of the complexes is defined as follows:Δ*E*_S_ = *E*(complexes) − [*E*(cation) + *E*(anion) + *E*(dimer)]

The interaction energies are corrected by the basis set superposition error (BSSE) and zero-point energy (ZPE). Thus, the corrected interaction energy Δ*E* can be calculated as follows:Δ*E* = Δ*E*_S_ + Δ*E*_BSSE_ + Δ*E*_ZPE_where Δ*E*_BSSE_ is the correction of BSSE and Δ*E*_ZPE_ is the correction of ZPE.

To further study the interactions among ILs EG and dimer, natural bond orbital (NBO) analysis^46^ was performed by NBO 3.1 program. Depending on the results of NBO analysis, the second-order perturbation stabilization energy *E*(2) with the delocalization of *i* → *j* is estimated as:
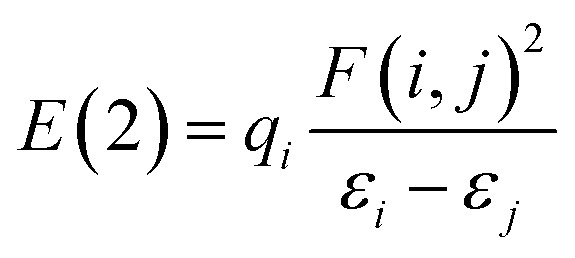


The optimized configurations of dimer-ILs were also illustrated on the basis of quantum theory of atoms in molecules (AIM) analysis.^47^ Reduced density gradient (RDG) function combined with sign(*λ*_2_)*ρ* was used to distinguish H-bond interactions from other weak interactions.^48^ All non-covalent interactions in the system are indicated in Fig. S2.† The topological properties were determined with Multiwfn program^49^ with the wave functions generated from the B3LYP-D3/6-311+G** results. RDG surfaces and a schematic graph of the electron transfer based on NBO analysis are plotted by VMD program.^50^

## 3. Results and discussion

### 3.1 Interaction between dimer and anions/cations

#### 3.1.1 Geometry and interaction energy of dimer and anions/cations

**Fig. 1:**
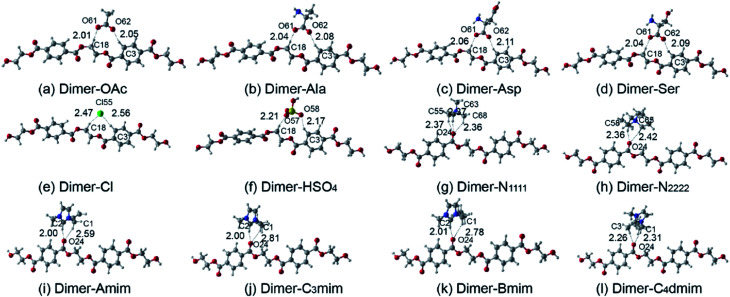
Optimized dimer-anion (a–f) and dimer-cation (g–l) conformers at the level of B3LYP/6-31++G**. H-bonds are indicated by dashed lines. Distances are in angstroms.

The structures of dimer and the isolated anions and cations are optimized at the B3LYP/6-31++G** level from different initial guesses (Fig. S3–S5†) and the most stable conformers dimer-OAc^−^, dimer-Ala^−^, dimer-Asp^−^, dimer-Ser^−^, dimer-Cl^−^, dimer-HSO_4_^−^, dimer-N_1111_^+^, dimer-N_2222_^+^, dimer-Amim^+^, dimer-C_3_mim^+^, dimer-Bmim^+^, and dimer-C_4_dmim^+^ are depicted in Fig. 1. The strongest interaction between dimer and anions appears when H-bonds are formed between the electronegative atoms (Cl, O) of anions and the carboxyl of dimer. Alternately, the strongest interaction between dimer and cations appears when H-bonds are formed between the C2–H of the imidazolium ring and the carbonyl oxygen of dimer. The formation of O–H⋯O and O–H⋯Cl H-bonds is determined if the distances between O⋯H and Cl⋯H are less than 2.72 and 2.95 Å (ref. 51), which are the sum of the van der Waals radii, respectively. The geometries of dimer and isolated anions/cations optimized at the B3LYP-D3/6-311+G** level are listed in Table S3.† The corresponding H-bond distances are labeled in Fig. 1 and the interaction energies corrected by BSSE are summarized in Table 1. Recently, Grimme *et al.* proposed the DFT-D3 study of some molecular crystals^52^ and Zahn's work in ionic liquids^53^ that may have reference values. The data computed by three levels, including the B3LYP/6-31++G**, B3LYP-D3/6-31++G** and B3LYP-D3/6-311+G**, are shown in Table 1. It is observed that the interaction energies calculated at B3LYP-D3/6-31++G** are nearly 10–30 kJ mol^−1^ higher than those calculated at B3LYP/6-31++G**, showing that D3 can consider the dispersion effects to some extent. As for the effects of dispersion in geometry, taking dimer-Oac^−^ as an example, the H-bond length of C3–H8⋯O62 and C18–H20⋯O61 optimized at B3LYP-D3/6-311+G** are 2.139 and 2.149 Å, respectively. This is slightly longer than the geometries optimized without dispersion. However, it does not affect the overall form of interaction. In addition, the previous basis B3LYP/6-31++G** may not be as accurate as B3LYP-D3/6-311+G** on consideration of dispersion interaction, which still gives a reasonable tendency consistent with experimental results.^32,39,41^

**Table 1:** Comparison of interaction energies BSSE (a.u.) and Δ*E* (kJ mol^−1^) at the B3LYP/6-31++G**, B3LYP-D3/6-31++G** and B3LYP-D3/6-311+G** levels of theory

Structure	B3LYP/6-31++G**		B3LYP-D3/6-31++G**		B3LYP-D3/6-311+G**	
	BSSE (a.u.)	Δ*E*	BSSE (a.u.)	Δ*E*	BSSE (a.u.)	Δ*E*
Dimer-OAc^−^	0.001242	−75.71	0.001576	−95.17	0.001564	−95.44
Dimer-Ala^−^	0.001102	−67.56	0.001761	−90.75	0.002127	−93.44
Dimer-Asp^−^	0.001144	−62.54	0.002928	−87.40	0.002962	−88.41
Dimer-Ser^−^	0.001167	−66.42	0.002040	−90.88	0.002082	−90.97
Dimer-Cl^−^	0.000368	−60.62	0.000391	−76.12	0.001051	−76.11
Dimer-HSO_4_^−^	0.002218	−50.84	0.002734	−78.96	0.002851	−73.44
Dimer-N_1111_^+^	0.000839	−42.57	0.000866	−61.26	0.000620	−63.34
Dimer-N_2222_^+^	0.000911	−28.03	0.001252	−49.21	0.001012	−50.73
Dimer-Amim^+^	0.001097	−42.10	0.001185	−57.82	0.000777	−59.79
Dimer-C_3_mim^+^	0.001030	−41.03	0.001280	−58.25	0.001048	−58.75
Dimer-Bmim^+^	0.001037	−40.55	0.001680	−52.67	0.001477	−54.90
Dimer-C_4_dmim^+^	0.000927	−29.49	0.001526	−52.19	0.001390	−52.65

From Fig. 1, the interaction of cations is more intensive at the oxygen of the carbonyl of PET, while anions are more likely to attack the hydrogen on the chain of PET. Besides, the anions can induce a more substantial reversal distortion in PET. In Table 1, comparing the interaction energies of these structures, it indicates that the Δ*E* of anions decreases in the order: OAc^−^ > Ala^−^ > Ser^−^ > Asp^−^ > Cl^−^ > HSO_4_^−^, with the sequence of cations being: N_1111_^+^ > Amim^+^ > C_3_mim^+^ > Bmim^+^ > C_4_dmim^+^ > N_2222_^+^. The interaction energies of dimer with anions are larger than those with cations. Anions may play more important roles than cations. Compared with the interaction data for amino acid anions, when the hydrogen of methyl is replaced by hydroxyl or carboxyl, the interaction becomes weak. The stronger the electronegativity of the replaced group is, the weaker the interaction will be. For the quaternary ammonium and imidazolium cations, the interactions decrease with the growth of the alkyl chain, but the change is not obvious for the imidazolium cations because it has little influence on charge distribution and the positive charge is always distributed around the imidazolium ring. When the hydrogen of C2 was replaced by methyl, the interaction was weaker. However, when the alkyl chain was replaced by the unsaturated hydrocarbon, the interaction was stronger. The results are consistent with the experimental data.^28,32^ It is speculated that when the side chain functional groups are introduced into the anions, the electronegativity of the carboxyl is reduced and in the cations the interaction is more related to the van der Waals interactions and steric effects. These rules may have a certain guiding significance for designing ILs catalysts.

#### 3.1.2 NBO analysis of dimer and anions/cations

The natural bond orbital (NBO) method is characterized by the H-bonds in terms of hyper-conjugative donor–acceptor interactions and has been used to study the bonding properties in these conformers.^46,54^ The main donor–acceptor interactions between dimer and anions/cations, as well as their second-order perturbation stabilization energies, are listed in Table S4.† The value of *E*(2) denotes the strength of the donor–acceptor interaction, and the larger the value is, the stronger the interaction will be.

**Fig. 2:**
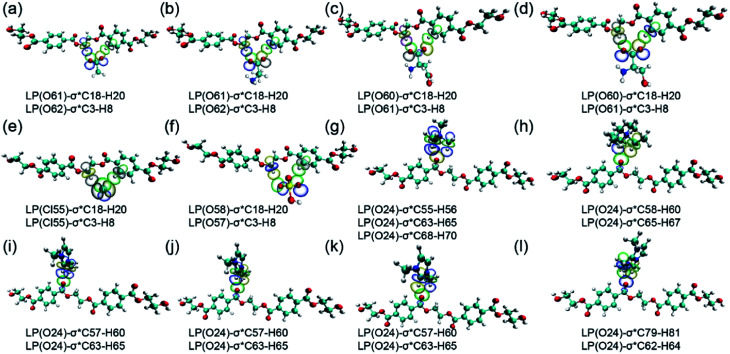
Schematic graph of (a) dimer-OAc, (b) dimer-Ala, (c) dimer-Asp, (d) dimer-Ser, (e) dimer-Cl, (f) dimer-HSO_4_, (g) dimer-N_1111_, (h) dimer-N_2222_, (i) dimer-Amim, (j) dimer-C_3_mim, (k) dimer-Bmim and (l) dimer-C_4_dmim electron transfer occurring from the lone pairs of O atoms to the anti-bonding orbital of the proton donor based on the NBO analysis.

The obvious and efficient overlaps are found between anti-bonding orbitals of dimer and lone-pair orbitals of anions, and between lone-pair orbitals of dimer and anti-bonding orbitals of cations. For dimer-OAc^−^, which contains two types of intramolecular H-bonds: C18-H20⋯O61 and C3-H8⋯O62, it is found that the *E*(2) values of LP O61 → σ*C18-H20 and LP O62 → σ*C3-H8 are 45.528 and 26.628 kJ mol^−1^, respectively. Compared with the *E*(2) data for strongest H-bonds formed in dimer-anions, it could be found that the order of formed H-bonds with dimer follows: OAc^−^ > Ala^−^ > Ser^−^ > Asp^−^. The largest *E*(2) (C2–H⋯O) values in the imidazolium cations are 35.274, 33.810, 26.040 and 12.642 kJ mol^−1^, corresponding to dimer-Amim^+^, dimer-C_3_mim^+^, dimer-Bmim^+^ and dimer-C_4_dmim^+^, respectively. The corresponding orbitals of some typical dimer-anions/cations are listed in Fig. 2. The length of the alkyl chain has little effect on the dimer-cations interaction, as can be seen from the small difference in *E*(2) energies. In addition, the PET is overturned by the action of anions. This verifies that anions play a key role in the glycolysis of PET.

#### 3.1.3 AIM analysis of dimer and anions/cations

In this part, atoms in molecules (AIM) theory is applied to investigate the bonding properties of the intermolecular interaction. Topology parameters, such as electron density (*ρ*_BCP_), laplacian of electron density (∇^2^*ρ*_BCP_), potential energy density (*V*(*r*)), and energy density (*H*_BCP_) at the bond critical points (BCPs) are often used to characterize the types of interactions.^55,56^ Espinosa *et al.* stated that for the H-bond [X–H⋯O (X = C, N, O)] the energy is half of the potential energy density *V*(*r*).^47^ Two topology criteria for the existence of H-bonds were proposed by Lipkowski.^57^ The electron density as well as the laplacian of the electron density for closed-shell interactions as H-bonds should be within the following ranges: *ρ*_BCP_ is in 0.002–0.034 a.u., and ∇^2^*ρ*_BCP_ is in 0.024–0.139 a.u. Rozas and his co-authors^58^ introduced a criterion to classify the strength of H-bonds in terms of ∇^2^*ρ*_BCP_ and *H*_BCP_. For weak H-bonds, both of the ∇^2^*ρ*_BCP_ and *H*_BCP_ are positive. For medium H-bonds, ∇^2^*ρ*_BCP_ is positive but *H*_BCP_ is negative. For the strong H-bonds, both of the ∇^2^*ρ*_BCP_ and *H*_BCP_ are negative.

In Table 2, it was found that the largest *ρ*(*r*) are 0.0248, 0.0232, 0.0223 and 0.0229 a.u., corresponding to dimer-OAc^−^, dimer-Ala^−^, dimer-Asp^−^ and dimer-Ser^−^, respectively. It indicates that the largest *ρ*(*r*) value in dimer-OAc^−^ is larger than that of other complexes. The H-bond formed with the interchain in the dimer-OAc^−^ is much stronger than that in the other anions. In the imidazolium cations, the largest *ρ*(*r*) are 0.0214, 0.0212, 0.0208 and 0.0129 a.u., corresponding to dimer-Amim^+^, dimer-C_3_mim^+^, dimer-Bmim^+^ and dimer-C_4_dmim^+^, respectively. It manifests that the interactions of anions with dimer are much stronger than those of cations. These results are also consistent with the conclusion of the H-bonds discussed above.

**Table 2:** The electron density (*ρ*_BCP_), laplacian of the electron density (∇^2^*ρ*_BCP_), potential energy density (*V*(*r*)), and energy density (*H*_BCP_) for dimer-anions/cations (a.u.)

Structure	H-bond	*ρ*_BCP_	∇^2^*ρ*_BCP_	*H*_BCP_ (10^−3^ a.u.)
Dimer-OAc^−^	C18–H20⋯O61	0.0248	0.0650	−2.349
	C3–H8⋯O62	0.0207	0.0608	−0.141
Dimer-Ala^−^	C18–H20⋯O61	0.0232	0.0609	−2.195
	C3–H8⋯O62	0.0197	0.0579	−0.167
Dimer-Asp^−^	C18–H20⋯O61	0.0223	0.0598	−1.812
	C3–H8⋯O62	0.0188	0.0547	−0.280
Dimer-Ser^−^	C18–H20⋯O60	0.0229	0.0614	−1.902
	C3–H8⋯O61	0.0196	0.0569	−0.305
Dimer-Cl^−^	C18–H20⋯Cl55	0.0162	0.0444	2.195
	C3–H8⋯Cl55	0.0134	0.0369	2.520
Dimer-HSO_4_^−^	C18–H19⋯O57	0.0163	0.0459	−0.635
	C3–H8⋯O58	0.0169	0.0500	−0.085
Dimer-N_1111_^+^	C68–H70⋯O24	0.0114	0.0381	1.858
	C55–H56⋯O24	0.0109	0.0374	2.107
	C63–H65⋯O24	0.0108	0.0371	2.080
Dimer-N_2222_^+^	C58–H60⋯O24	0.0108	0.0370	2.020
	C65–H67⋯O24	0.0042	0.0163	2.218
Dimer-Amim^+^	C57–H60⋯O24	0.0214	0.0720	2.350
	C68–H70⋯O24	0.0073	0.0264	2.331
Dimer-C_3_mim^+^	C57–H60⋯O24	0.0212	0.0716	2.338
	C63–H65⋯O24	0.0044	0.0172	2.278
Dimer-Bmim^+^	C57–H60⋯O24	0.0208	0.0706	2.269
	C69–H71⋯O24	0.0046	0.0180	2.312
Dimer-C_4_dmim^+^	C79–H81⋯O24	0.0129	0.0428	1.606
	C62–H64⋯O24	0.0116	0.0396	1.881

#### 3.1.4 RDG analysis of dimer and anions/cations

**Fig. 3:**
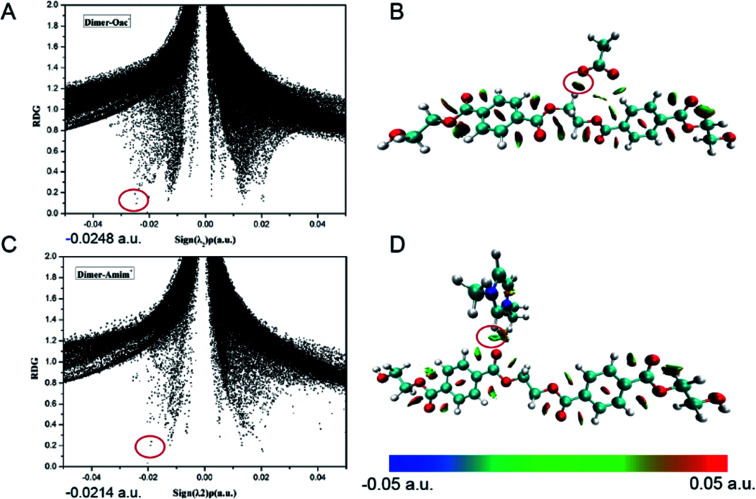
RDG isosurfaces (*s* = 0.6 a.u.) and surface plots of (A and B) dimer-OAc interaction and (C and D) dimer-Amim interaction. The isosurfaces are colored on a blue-green-red scale according to values of sign(*λ*_2_)*ρ*, ranging from −0.05 to 0.05 a.u. Red indicates strong attractive interactions and blue indicates strong nonbonded overlap.

RDG analysis is employed as another useful method to further study noncovalent interaction in this work. This method can also supply reliable data, similarly to the AIM method.^59^ The scatters of RDG and the electron density (*ρ*) multiplied by the sign of the second Hessian eigenvalue^48^ can make visualization and discrimination of weak interactions possible; visualizations of the inter- and intra-molecular weak interactions for dimer-OAc^−^ and dimer-Amim^+^ are shown in Fig. 3. The same analysis is suitable for other conformers formed between dimer and anions/cations, with the results shown in Fig. S6 and S7.† It can be seen that many RDG spikes in the scatter graph, as well as the spikes corresponding to H-bonding interactions, van der Waals interactions and steric effects, are shown as a function of sign(*λ*_2_)*ρ* from negative to positive values.

The spikes and disk-shaped blocks that represent the strongest H-bonds in dimer-anions/cations are marked by the red circles. As depicted in Fig. 3, every conformer has multiple spikes. The spike corresponding to the strongest H-bond of dimer-OAc^−^ is located at 0.0248 a.u. and is larger than that for the other anions. The regions and types of interactions between dimer and anions/cations can also be visualized by colored RDG isosurface. As shown in Fig. 3 and S7,† the corresponding region color of the disc-shaped block in dimer-OAc^−^ is dark blue, consistent with the light blue in other dimer-anions conformers. The spike corresponding to the strongest H-bond of dimer-N_1111_^+^ (0.0114 a.u.) is larger than that of dimer-N_2222_^+^ (0.0108 a.u.). At the same time, the spike corresponding to C57–H60⋯O24 of dimer-Amim^+^ is located at 0.0214 a.u., which is larger than other imidazolium cations. Meanwhile, we speculate that the length of the alkyl chain cannot affect the interaction significantly because it influences the charge distribution and the positive charges are always distributed around the imidazolium ring. The results are consistent with geometry, NBO and AIM analyses.

### 3.2 Interaction between dimer and ion pairs

#### 3.2.1 Geometry and interaction energy

**Fig. 4:**
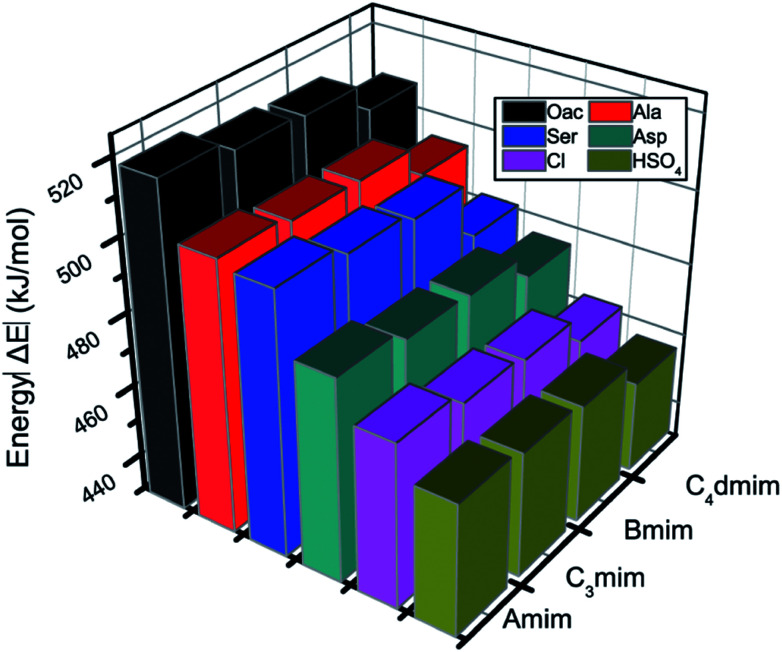
Interaction energy comparison of structures for dimer-ion pairs interaction.

It is of great importance to study the structural behavior between the model compound and ion pairs, which is essential to reveal the degradation mechanism. The interaction energies of 24 possible conformers (Fig. S8†) are compared in Fig. 4, wherein it is observed that increasing the length of the alkyl chain results in decreasing of interaction energies between dimer and ion pairs. Referring to the Δ*E*_D-C-A_ data in Table S5,† the binding energies for ion pairs are −415.47, −412.32, −410.82 and −397.82 kJ mol^−1^ at the B3LYP-D3/6-311+G** level for AmimOAc, C_3_mimOAc, BmimOAc and C_4_dmimOAc, respectively. The interaction energies between anions and cations will decrease with the increasing alkyl chain length of the substituent. This rule is also applied for other amino acid, HSO_4_ and Cl anions-typed ILs and the similar trend is obtained. The dimer-AmimOAc system has relatively higher binding energy, −518.64 kJ mol^−1^, while the binding energy in the other three OAc-based ILs are −515.28 kJ mol^−1^ (C_3_mimOAc), −514.23 kJ mol^−1^ (BmimOAc) and −506.32 kJ mol^−1^ (C_4_dmimOAc). It is found that the strongest interaction with PET is from the Amim-based ILs. The elongation of alkyl chain leads to the increase of the ion volume, promoting charge distribution to a greater extent, which leads to a decline of binding energies interaction between PET and ion pairs. Because the anion plays a critical role in the conformers and energy, the following natural bond orbital (NBO) analysis, atoms in molecules (AIM) theory, and reduced density gradient (RDG) analysis are performed for structures of the dimer-AmimOAc, dimer-AmimAla, dimer-AmimAsp, dimer-AmimSer, dimer-AmimCl and dimer-AmimHSO_4_ to specifically investigate the H-bonds formed in the system.

**Fig. 5:**
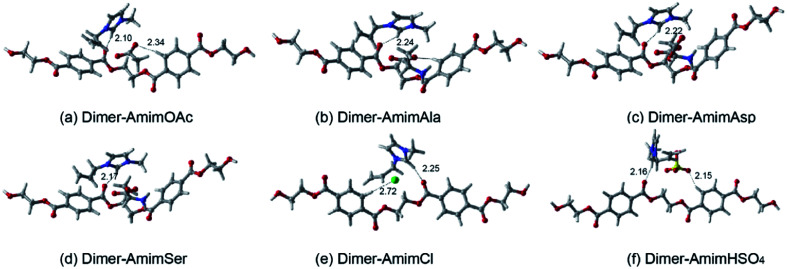
Optimized structures of interaction between dimer and Amim-based ILs (a–f) at the B3LYP-D3/6-311+G** level and H-bonds are indicated by dashed lines.

The interaction between dimer and the corresponding Amim based ILs conformers are depicted in Fig. 5. The corresponding interaction energies at different levels are summarized in Table 3. As is shown in Fig. 5(a), the calculated C18–H20⋯O80, C3–H8⋯O81 and C57–H60⋯O24 distances are 2.71, 2.34 and 2.10 Å, respectively, which are both remarkably shorter than the summation (2.72 Å) of the van der Waals radii of O and H atoms. The C18–H20⋯O80, C3–H8⋯O81 and C57–H60⋯O24 angles are 170.75°, 164.31° and 147.89°, respectively. These data indicate the formation of C18–H20⋯O80, C3–H8⋯O81 and C57–H60⋯O24 H-bonds between dimer and AmimOAc. The other conformers of dimer with Amim-based ILs have similar results, forming H bonds with the interchain, benzene ring and the oxygen of carbonyl group of dimer.

**Table 3:** Comparison of interaction energies Δ*E* (kJ mol^−1^) at the B3LYP/6-31++G**, B3LYP-D3/6-31++G** and B3LYP-D3/6-311+G** levels of theory. (D-dimer, C-cation, A-anion, CA-ion pairs) Δ*E*_CA_ = *E*_CA_ − *E*_C_ − *E*_A_, Δ*E*_CA-D_ = *E*C_A-D_ − *E*_CA_ − *E*_D_, Δ*E*_C-A-D_ = *E*_CA-D_ − *E*_C_ − *E*_A_ − *E*_D_

Structures	B3LYP/6-31++G**			B3LYP-D3/6-31++G**			B3LYP-D3/6-311+G**		
	Δ*E*_1,CA_	Δ*E*_1,CA-D_	Δ*E*_1,C-A-D_	Δ*E*_2,CA_	Δ*E*_2,CA-D_	Δ*E*_2,C-A-D_	Δ*E*_3,CA_	Δ*E*_3,CA-D_	Δ*E*_3,C-A-D_
Dimer-AmimOAc	−390.03	−44.98	−435.01	−415.01	−101.52	−516.53	−415.47	−103.18	−518.64
Dimer-AmimAla	−388.54	−42.79	−431.33	−416.49	−84.14	−500.63	−418.58	−85.41	−503.99
Dimer-AmimAsp	−372.03	−40.13	−412.16	−397.75	−100.82	−498.58	−399.24	−102.66	−501.90
Dimer-AmimSer	−371.96	−43.18	−415.14	−381.37	−101.07	−482.44	−382.61	−102.84	−485.45
Dimer-AmimCl	−351.53	−42.88	−394.41	−386.74	−72.50	−460.65	−391.48	−74.57	−466.05
Dimer-AmimHSO_4_	−355.12	−41.40	−396.52	−387.44	−73.21	−459.24	−391.04	−83.96	−475.00

As described before, the interactions of cations are more intensive at the oxygen of the carbonyl group of PET while anions are likely to attack the hydrogen on the chain of PET and can also make PET overturn. Besides, the binding energy for dimer-anions and dimer-cations at the B3LYP/6-31++G** level can well show the H-bonds in the systems. When there is only one ion (Section 3.1), the electrostatic interactions play a key role in the system. Grimme *et al.*^44^ demonstrated that B3LYP-D3 is an ideal choice to investigate large molecular systems as it takes the dispersion interaction into account to improve its capability for noncovalent interaction. The changes in H-bond lengths and angles are listed in Table S6,† which were used in the improvement of the B3LYP-D3/6-311+G** level. Three different calculation levels have been calculated and are listed in Table 3; we compared the interaction energies of dimer and Amim-based ILs conformers calculated at the B3LYP/6-31++G**, B3LYP-D3/6-31++G** and B3LYP-D3/6-311+G** levels of theory. It could be found that the interaction energy sequence of Δ*E*_1,C-A-D_ follows the order: AmimOAc > AmimAla > AmimSer > AmimAsp > AmimHSO_4_ > AmimCl. We further consider the π–π stacking effects on the ILs-PET systems by reoptimizing those geometries at the B3LYP-D3/6-311+G** level, which will give a good simulation of the total energies. The interaction of ion pairs (Δ*E*_2,CA_) is about 30–60 kJ mol^−1^ higher than Δ*E*_1,CA_, showing that the D3 term can well consider the π stacking interaction. Owing to the big proportion of electrostatic interaction in ILs, the data of Δ*E*_2,CA_ and Δ*E*_1,CA_ do not have such a great difference. However, it is clear from the data that the amino acid ILs are better than sulfuric acid and halogen in the degradation of PET. In the amino acid anions, when the hydrogen of methyl is replaced by hydroxyl or carboxyl, the interaction becomes weak. The stronger the electronegativity of the replaced group, the weaker the interaction will be. The result is consistent with the experimental data.^28,32^ Therefore, it can be speculated that both intermolecular H-bonds and π-stacking interactions are expected to be a key factors resulting in the degradation of PET and this rule may provide some basic aids to design an efficient ionic liquid catalyst.

#### 3.2.2 NBO analysis of dimer-ILs

**Fig. 6:**
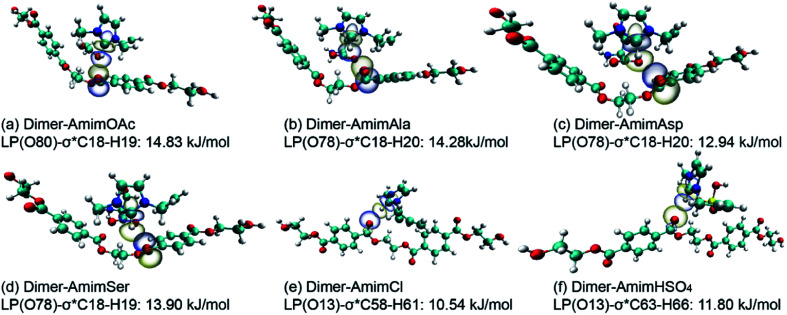
Natural bond orbital interaction in (a) dimer-AmimOAc, (b) dimer-AmimAla, (c) dimer-AmimAsp, (d) dimer-AmimSer, (e) dimer-AmimCl and (f) dimer-AmimHSO_4_ conformers calculated at the B3LYP-D3/6-311+G** level.

Further analysis of dimer and ionic pair interactions in complexes of interest is performed by second-order perturbation theory. The dominant effect is not only the H-bonds between dimer and anions but also the π-stacking interaction that are found here. The most important acceptor–donor interactions and their second-order perturbation stabilization energies are listed in Table S7.† Taking dimer-AmimOAc as an example, one can see the maximum values of *E*(2) of LP(O80) → σ* C18–H19 (14.83 kJ mol^−1^) and π C25–C26 → σ*C63–H65 (2.89 kJ mol^−1^), which means the H-bonds are stronger than π-stacking interactions. The largest interaction orbitals corresponding to Fig. 6(a) are symmetrical matching with the largest overlap. There are three kinds of H-bonds formed with dimer-AmimOAc, while the C18–H19⋯O80 H-bond plays a critical role in the degradation of PET. The maximum values of *E*(2) in amino acid anions are LP (O78) → σ* C18–H20 (14.28 kJ mol^−1^), LP(O78) → σ* C18–H20 (12.94 kJ mol^−1^), and LP(O78) → σ* C18–H20 (13.90 kJ mol^−1^). It can be concluded that when the side chain is replaced by a hydroxyl or carboxyl functional group the interaction becomes weak.

#### 3.2.3 AIM analysis of dimer-ILs

In order to reveal the interaction between the dimer model compound and ion pairs, AIM analysis is employed to verify the intermolecular interactions. As shown in Table 4, the values of *ρ*_BCP_, ∇^2^*ρ*_BCP_ and *H*_BCP_ are obtained from AIM calculations. The positive *ρ*_BCP_ values validated that there are electrostatic interactions between dimer and ion pairs in each structure. The negative value of *H*_BCP_ suggests that the H-bonds have the covalent characteristic and positive values of *H*_BCP_ suggest that the H-bonds have the electrostatic characteristic in the dimer-ionic pairs. Generally, the greater of the value of *ρ*_BCP_ means the shorter distance, and can reflect the intensity of H-bond.^60^ For most H-bonds analyzed here, both of the *ρ*_BCP_ and ∇^2^*ρ*_BCP_ values lie in the relative proposed ranges. The *ρ*_BCP_ of C18–H19⋯O80 and C3–H8⋯O81 in dimer-AmimOAc are 0.0172 and 0.0143 a.u., respectively. This result is in agreement with the bond length of C18–H19⋯O80 (2.196 Å), which is shorter than that of C3–H8⋯O81 (2.236 Å). For the dimer-AmimOAc conformer, *ρ*_BCP_ and ∇^2^*ρ*_BCP_ at BCPs confirm that the H-bonds formed between dimer and OAc are stronger than those for dimer and Amim. The above results show that the interaction of anion is stronger than that of cation and the H-bonds formed with the inter-chain play a more critical role in the degradation of PET.

**Table 4:** The electron density (*ρ*_BCP_), laplacian of the electron density (∇^2^*ρ*_BCP_), potential energy density (*V*(*r*)), and energy density (*H*_BCP_) for dimer-ILs (a.u.)

Structure	H-bond	*ρ*_BCP_	∇^2^*ρ*_BCP_	*H*_BCP_ (10^−3^ a.u.)
Dimer-AmimOAc	C18–H19⋯O80	0.0172	0.0460	−0.634
	C3–H8⋯O81	0.0143	0.0396	−0.090
	C57–H60⋯O24	0.0147	0.0452	0.293
Dimer-AmimAla	C18–H19⋯O78	0.0164	0.0418	−0.618
	C3–H8⋯O79	0.0142	0.0394	−0.092
	C57–H60⋯O24	0.0146	0.0456	0.274
Dimer-AmimAsp	C18–H19⋯O78	0.0161	0.0413	−0.564
	C3–H8⋯O79	0.0132	0.0372	−0.089
	C57–H60⋯O24	0.0150	0.0468	0.266
Dimer-AmimSer	C18–H19⋯O80	0.0163	0.0419	−0.615
	C3–H8⋯O81	0.0130	0.0365	−0.048
	C57–H60⋯O24	0.0156	0.0481	0.196
Dimer-AmimCl	C15–H16⋯Cl55	0.0129	0.0357	0.918
	C26–H29⋯Cl55	0.0075	0.0216	0.948
	C58–H61⋯O13	0.0127	0.0411	0.488
Dimer-AmimHSO_4_	C15–H16⋯O56	0.0136	0.0408	0.084
	C26–H29⋯O58	0.0136	0.0382	−0.089
	C63–H66⋯O13	0.0099	0.0365	1.075

#### 3.2.4 RDG analysis of dimer-ILs

**Fig. 7:**
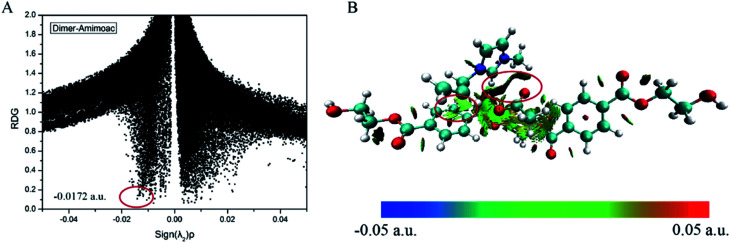
(A) RDG scatter plots and (B) isosurfaces (*s* = 0.6 a.u.) of dimer-AmimOAc conformer. The surfaces are colored on a blue-green-red scale according to values of sign(*λ*_2_)*ρ*, ranging from −0.05 to 0.05 a.u. Red indicates strong attractive interactions and green indicates strong nonbonded overlap.

The noncovalent interactions in this part revealed strong attraction, van der Waals effect and steric repulsion in the system. Reduced density gradient and the electron density multiplied by the sign of the second Hessian eigenvalue are plotted by scatter graph; the visualization of these inter- and intra-molecular weak interactions is shown in Fig. 7. Because of the similarity among the four conformers, dimer-AmimOAc is taken as an example. The sign(*λ*_2_)*ρ* ranges from −0.05 to 0.05, and the RDG ranges from 0 to 2.0. The strongest interaction formed dimer with ILs is around the inter-chain of PET, which makes the preparation for the glycolysis of PET. Spikes at the position −0.0172 a.u. marked by red circles indicate the strongest H-bond between the anion and cation of ILs. Other large green surfaces can be classified with π-stacking interaction between the benzene ring and the cation. The blue region denotes van der Waals interaction and weak H-bond interaction, the green region indicates that van der Waals interaction and steric effect coexist at the same time, while the red region shows the steric effect. Hence, we can make the conclusion that there are both π-stacking and H-bonds interactions in the dimer-AmimOAc system. The largest region with green and breen color plotted with red circle is found in dimer-AmimOAc, which is in agreement with the fact that dimer-AmimOAc has the largest value of interaction energy.

As depicted in Fig. 7 and S9,† every conformer has multiple spikes. The electron densities of dimer-AmimOAc, dimer-AmimAla, dimer-AmimAsp and dimer-AmimSer are 0.0172, 0.0164, 0.0161 and 0.0163 a.u., respectively. The interactions of dimer and Amim-based ILs decreases when the anion's functional group is replaced by hydroxyl or carboxyl. Owing to the steric effect of imidazolium cations, the H-bonds of cations are weaker than those of anions. These results are also consistent with the conclusion of H-bonds discussed above.

### 3.3 Interaction between EG and ion pairs

**Fig. 8:**
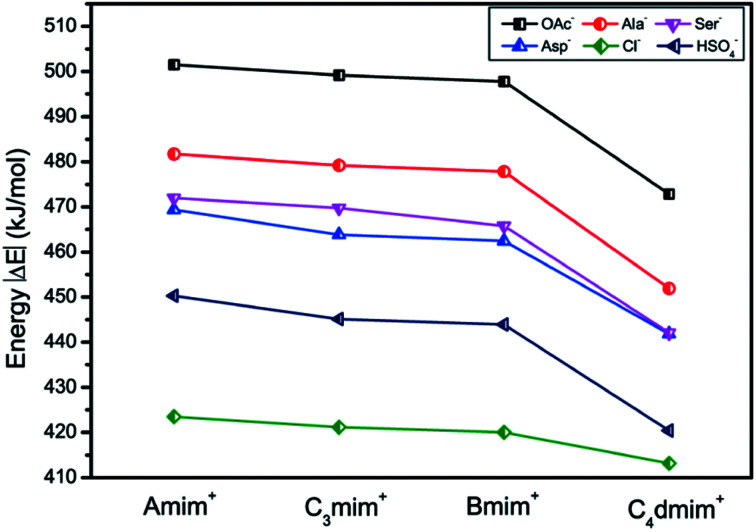
Interaction energy comparison of structures for EG-ion pairs interaction.

In the degradation of PET catalyzed by ILs, the system contains three kinds of compounds; ILs will not only interact with PET but also with EG. The interaction energies (Table S8†) of 24 possible conformers are compared in Fig. 8 and the structures of EG-ion pairs (Fig. S10†), and it could be seen that the interaction energies trends between EG-ion pairs and dimer-ion pairs are similar. The coordination of EG with ILs *via* H-bonds will promote the degradation of PET. The NPA changes and the O–H bond length of EG have been calculated, which are shown in Fig. S11 and S12,† respectively. The changes in bond length for EG while it interacts with ILs are listed in Table S9.† The interactions between EG and ILs makes the electronegativity of the oxygen of the hydroxyl in EG stronger. Besides, the length of the O–H bond of the hydroxyl group in EG becomes longer than those before interaction, which causes the hydrogen to be lost more easily. Therefore, the coordination structure of EG with ILs can activate the hydroxyl in EG to enhance the glycolysis of PET and finally facilitate the attack of oxygen on the carbon of the ester group in PET.

## 4. Conclusions

ILs have been used as environmentally friendly catalysts in the degradation of PET under mild conditions because of their adjustable physical and chemical properties. To investigate the interaction of PET/ILs/EG and the role of different anions/cations in the system, 24 kinds of anions/cations are studied in our work while dimer is chosen as the model of PET. It was found that H-bonds are formed in the system and play a critical role in the degradation of PET by DFT calculation. The comparison of interaction energies indicates that the anions play a critical role in the degradation of PET. In addition, H-bond interactions are particularly investigated and characterized by NBO analysis, AIM analysis and RDG method to evaluate the strength of the interactions. These intermolecular H-bonds and π-stacking interaction are expected to be a key factor resulting in the degradation of PET. The interactions of cations are more intensive at the oxygen of the carbonyl of PET. When the hydrogen of methyl is replaced by hydroxyl or carboxyl in amino acid anions, the interaction becomes weak. The stronger the electronegativity of the replaced group, the weaker the interaction will be. For the quaternary ammonium and imidazolium cations, the interactions decrease along with the growth of the alkyl chain. In the imidazolium cations, the change is not obvious owing to the small change of the alkyl chain. When the hydrogen of C2 was replaced by methyl, the interaction was weaker. Furthermore, when the alkyl chain is replaced by the unsaturated hydrocarbon, the interaction can be stronger. These rules may have a certain guiding significance for designing highly efficient ILs catalysts.

## Conflicts of interest

There are no conflicts to declare.

## Supplementary Material

RA-008-C7RA13173A-s001
