# Supplementary material for: Theoretical studies on glycolysis of poly(ethylene terephthalate) in ionic liquids
Source: RSC Adv. 2018 Feb 21;8(15):8209–19. doi: 10.1039/c7ra13173a (PMC9078552; doi:10.1039/c7ra13173a)
Supplement: RA-008-C7RA13173A-s001 [file RA-008-C7RA13173A-s001.pdf]

Electronic Supplementary Material (ESI) for RSC Advances.
This journal is © The Royal Society of Chemistry 2018

Supporting Information

Theoretical Studies on Glycolysis of Poly (ethylene terephthalate) in Ionic Liquids

Zhaoyang Ju[a,b], Weihua Xiao[a], Xingmei Lu[b], Xiaomin Liu[b], Xiaoqian Yao[b*], Xiaochun Zhang[b] and Suojiang Zhang[b*]

[a] *College of Engineering, China Agricultural University, Beijing 100083, P. R. China*

[b] *Key Laboratory of Green Process and Engineering, Beijing Key Laboratory of Ionic Liquids Clean Process, State Key Laboratory of Multiphase Complex Systems, Institute of Process Engineering, Chinese Academy of Sciences, Beijing 100190, PR China*

As predicting the reactive site at molecular surface is of theoretical and practical significance, many prediction methods based on the electrochemical structure of reactants have been proposed such as quantitative molecular surface analysis of electronic potential (ESP). The electrostatic potential surfaces for the most stable geometries of cation, anion, EG and dimer are given in Fig. S1. As is shown, the highly negative region of anion is on the electronegative carboxyl group and the electrostatic potential values of OAc^-, Ala^-, Asp^-, and Ser^- are -0.247, -0.236, -0.227, -0.233 a.u., respectively. Because of the symmetrical structure in the N_{1111}^+ and N_{2222}^+, the highly positive areas are 0.162 and 0.196 a.u. around the nitrogen. In the imidazolium cations, the highly positive of $Amim^+$, C_3mim^+, $Bmim^+$, C_4dmim^+ are 0.193, 0.192, 0.191 and 0.173 a.u.,respectively. The ESP value in dimer which around the electronegative carbonyl group is -0.051 a.u. The most positive and negative ESP values in EG are 0.072, -0.050 a.u, respectively.

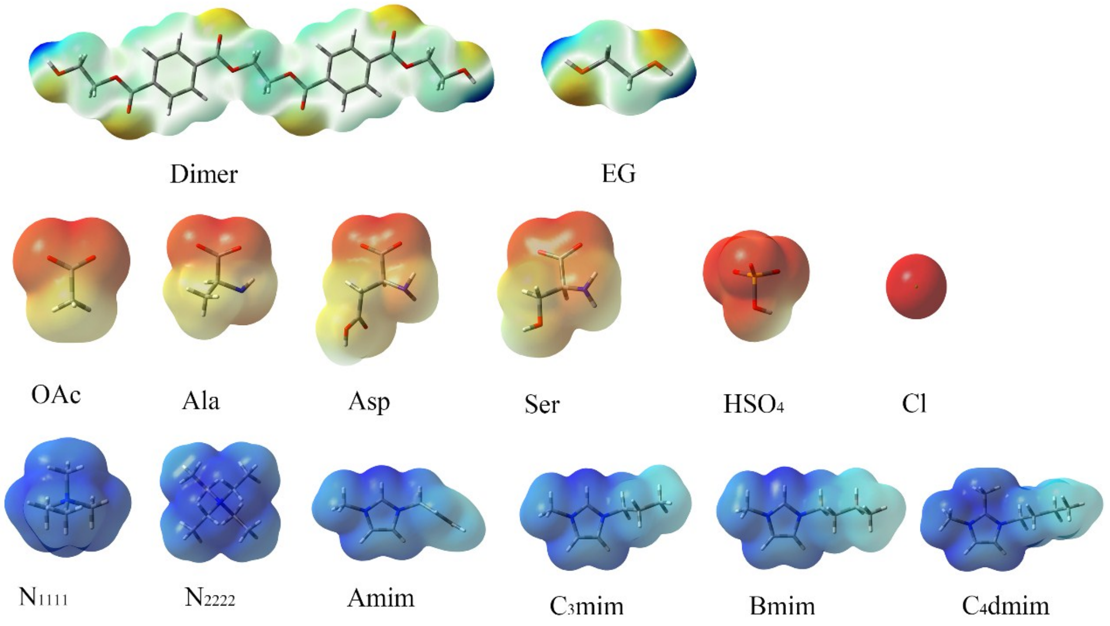

Fig. S1 The electrostatic potential surface of Dimer, EG anions and cations.

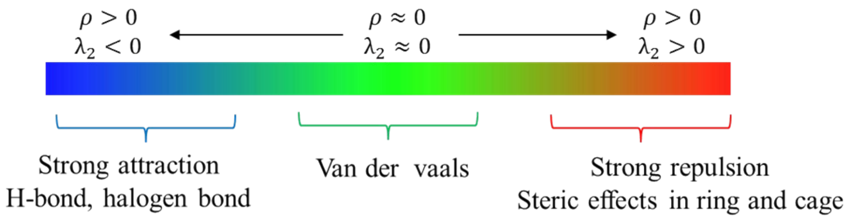

Fig. S2 Clarification of non-bonding interaction.

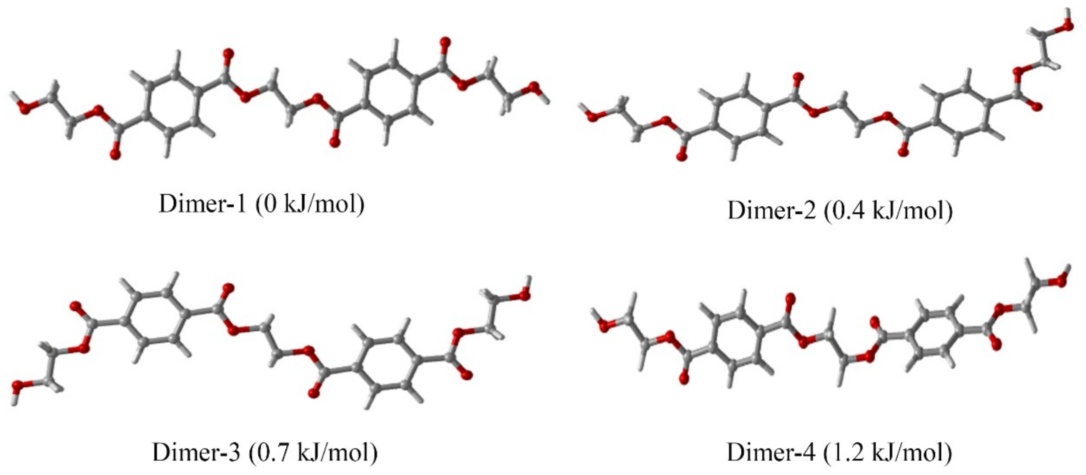

Fig. S3 Conformational isomers of Dimer model compound, and the relative energies to Dimer-1 are labeled in parentheses.

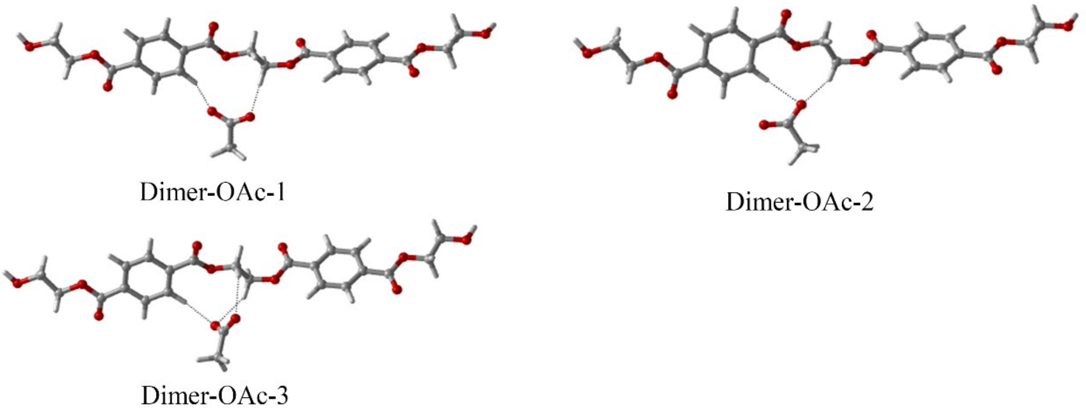

Fig. S4 The optimized configurations of Dimer-OAc-n (n=1-7) at B3LYP/6-31++G** level. The hydrogen bonds are indicated by dashed lines

Table S1 Hydrogen bonds and interaction energy between model compound and OAc

Structures	H bonding	Length[Å]	Angle [°]	ΔE (kJ/mol)	ΔΔE (kJ/mol)
Dimer-OAc-1	C3-H8···O62	2.054	160.92	-78.97	0
	C18-H20···O61	2.012	175.60		
Der-OAc-2	C3-H8···O62	2.279	168.29	-72.81	6.16
	C18-H20···O62	2.085	155.62		
Dimer-OAc-3	C3-H8···O62	2.214	164.20	-75.94	3.03
	C18-H20···O62	2.325	151.06		
	C15-H17···O61	2.450	122.81		

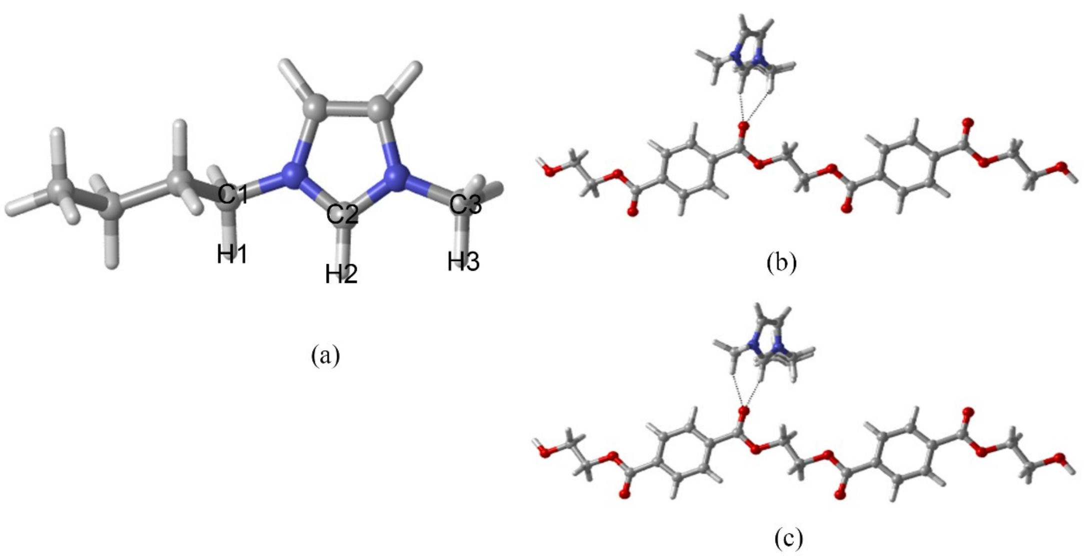

Fig. S5 The most stable structures of Dimer-Bmim⁺ model compound. (a) the structure of Bmim⁺ (b) indicate the interaction of Dimer-Bmim⁺ with C3-H3…O13 and C2-H2…O13 H-bonds (c) means the interaction of Dimer-Bmim⁺ with C1-H1…O13 and C2-H2…O13 H-bonds; All structures were optimized at the B3LYP/6-31++G** level.

Table S2 Hydrogen bonds and interaction energy between model compound and Bmim⁺

Structures	H bonding	Length[Å]	Angle [°]	ΔE (kJ/mol)	ΔΔE (kJ/mol)
(b)	C2-H2···O13	2.002	160.00	-43.27	0
	C1-H1···O13	2.785	142.43		
(c)	C2-H2···O13	2.059	150.78	-42.17	1.1
	C3-H3···O13	2.692	146.84		

Table S3 H-bond lengths and angles of Dimer-anions/cations conformers at the level of B3LYP/6-31++G** and B3LYP-D3/6-311+G**

	B3LYP/6-31++G**			B3LYP-D3/6-311+G**		
**Structure**	**H-bond**	**Length(Å)**	**Angle (°)**	**H-bond**	**Length(Å)**	**Angle (°)**
Dimer-OAc	C3-H8···O62	2.054	160.92	C3-H8···O62	2.139	153.56
	C18-H20···O61	2.012	175.60	C18-H20···O61	2.149	159.71
Dimer-Ala	C3-H8···O62	2.077	160.99	C3-H8···O62	2.141	156.03
	C18-H20···O61	2.044	177.27	C18-H20···O61	2.175	176.39
Dimer-Asp	C3-H8···O62	2.106	158.86	C3-H8···O62	2.098	147.98
	C18-H20···O61	2.056	176.81	C18-H20···O61	2.208	158.54
Dimer-Ser	C3-H8···O61	2.086	158.86	C3-H8···O61	2.285	146.25
	C18-H20···O60	2.043	176.81	C18-H20···O60	2.109	165.87
Dimer-Cl	C3-H8···Cl55	2.555	166.21	C3-H8···Cl55	2.589	152.58
	C18-H20···Cl55	2.467	159.61	C18-H20···Cl55	2.533	140.19
Dimer-HSO_4	C3-H8···O58	2.167	151.95	C3-H8···O58	2.077	152.33
	C18-H20···O57	2.214	166.05	C18-H20···O57	2.276	146.24
Dimer-N_{1111}	C55-H56···O24	2.367	145.55	C55-H56···O24	2.282	143.48
	C68-H70···O24	2.357	146.02	C68-H70···O24	2.265	144.21

	C63-H65···O24	2.372	145.37	C63-H65···O24	2.286	143.38
Dimer-N_{2222}	C65-H67···O24	2.419	154.61	C65-H67···O24	2.306	160.82
	C58-H60···O24	2.359	156.76	C58-H60···O24	2.355	155.31
Dimer-Amim	C2-H2···O24	2.001	155.10	C2-H2···O24	2.008	147.51
	C1-H1···O24	2.593	147.32	C1-H1···O24	2.351	149.49
Dimer-C_3mim	C2-H2···O24	1.996	160.84	C2-H2···O24	2.029	145.74
	C1-H1···O24	2.813	141.46	C1-H1···O24	2.385	141.48
Dimer-Bmim	C2-H2···O24	2.002	160.00	C2-H2···O24	2.055	143.31
	C1-H1···O24	2.785	142.43	C1-H1···O24	2.544	120.43
Dimer-C_4dmim	C3-H3···O24	2.258	171.25	C3-H3···O24	2.145	173.07
	C1-H1···O24	2.310	171.72	C1-H1···O24	2.359	142.59

Table S4 The main electron donor-acceptor interactions in the Dimer-anions/cations conformers and their second-order perturbation stabilization energies (E(2)).

**Structure**	**Donor (i)**	**Acceptor (j)**	**E(2) kJ/mol**	**ε(i)-ε(j)**	**F(i,j)**
Dimer-OAc^-	LP O61	σ* C18-H20	45.528	0.70	0.080
	LP O62	σ* C3-H8	26.628	0.71	0.061
Dimer-Ala^-	LP O61	σ* C18-H20	31.248	0.70	0.066
	LP O62	σ* C3-H8	24.150	0.71	0.058
Dimer-Asp^-	LP O60	σ* C18-H20	26.544	0.70	0.061
	LP O61	σ* C3-H8	21.756	1.14	0.069
Dimer-Ser^-	LP O60	σ* C18-H20	29.148	0.70	0.064
	LP O61	σ* C3-H8	22.386	1.13	0.069
Dimer-Cl^-	LP Cl55	σ* C18-H20	28.560	0.73	0.063
	LP Cl55	σ* C3-H8	20.034	0.67	0.050
Dimer-HSO_4^-	LP O58	σ* C18-H20	9.786	0.73	0.038
	LP O57	σ* C3-H8	6.132	0.76	0.031
Dimer-N_{1111}^+	LP O24	σ* C55-H56	7.308	1.14	0.040
	LP O24	σ* C63-H65	8.570	1.14	0.040
	LP O24	σ* C68-H70	9.618	1.14	0.046
Dimer-N_{2222}^+	LP O24	σ* C58-H60	8.946	1.15	0.044
	LP O24	σ* C65-H67	7.098	1.14	0.039
Dimer-$Amim^+$	LP O24	σ* C57-H60	35.274	1.16	0.087
	LP O24	σ* C63-H65	2.688	1.13	0.024
Dimer-C_3mim^+	LP O24	σ* C57-H60	33.810	1.15	0.086
	LP O24	σ* C63-H65	1.008	1.13	0.015
Dimer-$Bmim^+$	LP O24	σ* C57-H60	26.040	1.14	0.075
	LP O24	σ* C63-H65	4.578	1.15	0.032
Dimer-C_4dmim^+	LP O24	σ* C79-H81	12.642	1.14	0.053
	LP O24	σ* C62-H64	11.214	1.13	0.050

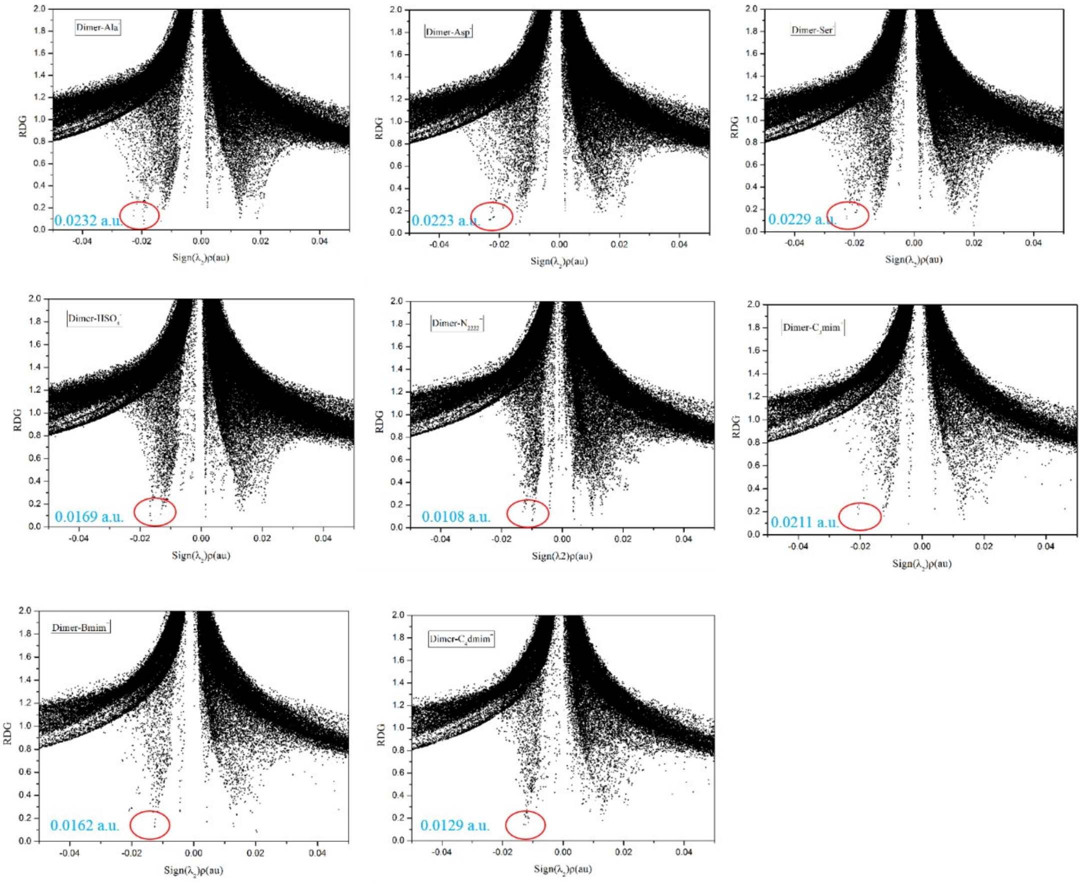

Fig. S6 Scatters of reduced density gradient versus the electron density multiplied by the second Hessian matrix sign(λ_2)ρ for conformers of interest calculated at B3LYP/6-31++G** level

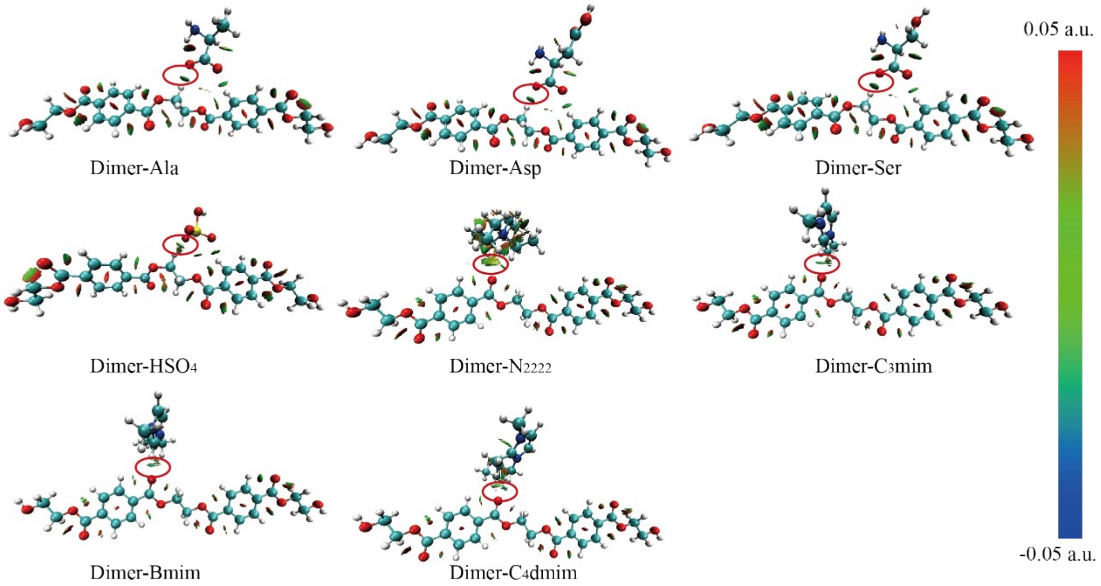

Fig. S7 Reduced density gradient isosurfaces (s=0.6 a.u.) for selected conformers calculated at B3LYP/6-31++G** level. The surfaces colored on a red-green blue scale according to values of sign(λ_2)ρ. Red indicates strong attractive interactions, and blue indicates strong nonbonded overlap

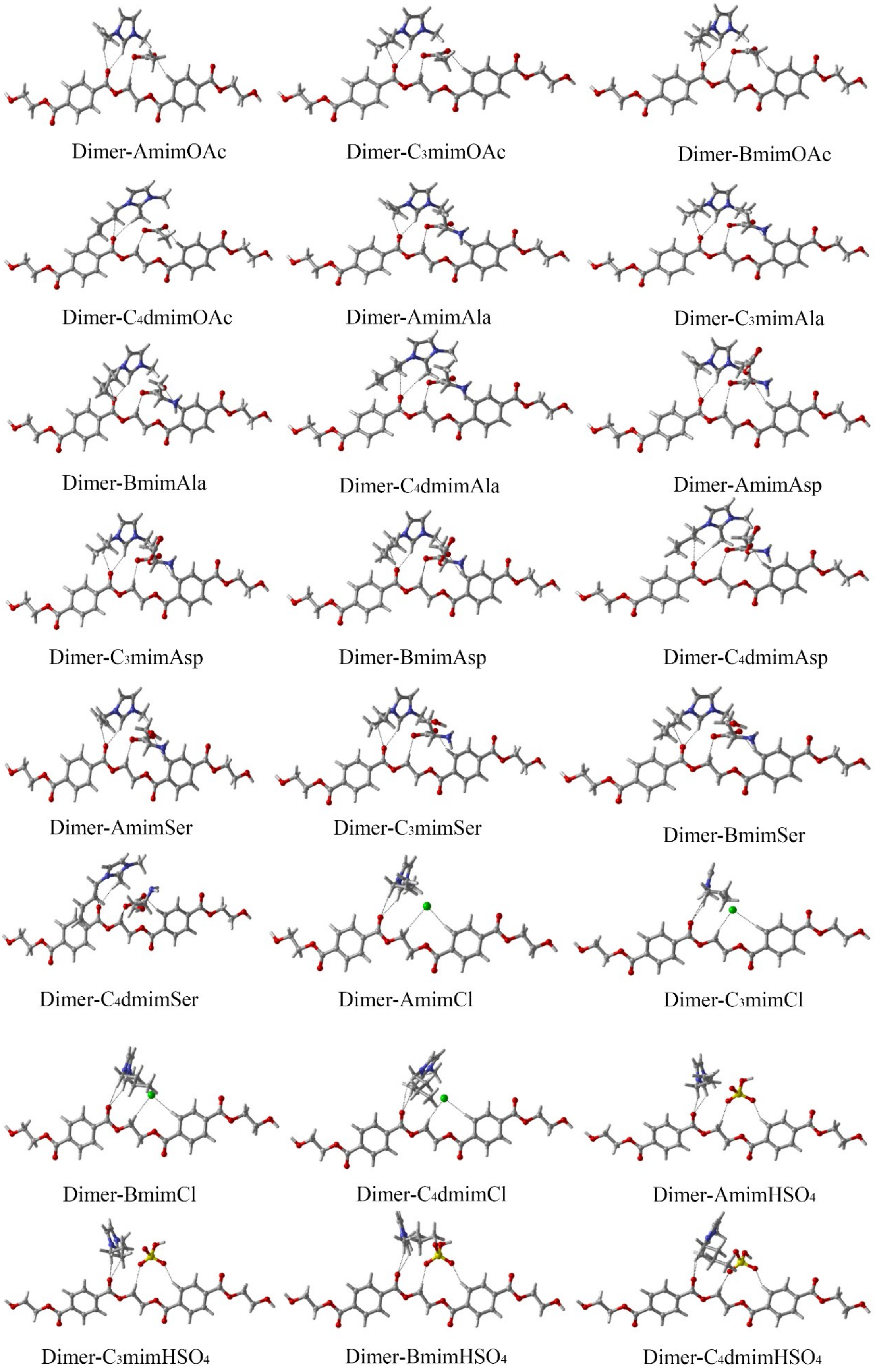

Fig. S8 Results of interaction energy calculation for the 24 kinds of Dimer model compound and ionic

liquids

Table S5 Calculated interaction energy at the B3LYP-D3/6-311+G** level of theory. (D-dimer, C-cation, A-anion, CA-ion pairs)

Entry	Structures	ΔE $_{CA}$(kJ/mol)	ΔE$_{D-CA}$ (kJ/mol)	ΔE$_{D-C-A}$ (kJ/mol)
1	Dimer-AmimOAc	-415.47	-103.18	-518.64
2	Dimer-C_3mimOAc	-412.32	-102.96	-515.28
3	Dimer-BmimOAc	-410.82	-103.41	-514.23
4	Dimer-C_4dmimOAc	-397.82	-108.51	-506.32
5	Dimer-AmimAla	-418.58	-85.41	-503.99
6	Dimer-C_3mimAla	-418.04	-84.10	-502.14
7	Dimer-BmimAla	-411.65	-90.11	-501.76
8	Dimer-C_4dmimAla	-408.39	-84.60	-492.99
9	Dimer-AmimAsp	-399.24	-102.66	-501.90
10	Dimer-C_3mimAsp	-399.84	-98.84	-498.68
11	Dimer-BmimAsp	-398.67	-97.71	-496.38
12	Dimer-C_4dmimAsp	-393.03	-87.68	-480.71
13	Dimer-AmimSer	-382.61	-102.84	-485.45
14	Dimer-C_3mimSer	-378.65	-103.36	-482.01
15	Dimer-BmimSer	-377.98	-103.28	-481.26
16	Dimer-C_4dmimSer	-384.77	-89.58	-474.35
17	Dimer-AmimHSO_4	-391.04	-83.96	-475.00
18	Dimer-C_3mimHSO_4	-381.13	-90.02	-471.15
19	Dimer-BmimHSO_4	-381.22	-88.04	-469.26
20	Dimer-C_4dmimHSO_4	-370.00	-91.38	-461.38
21	Dimer-AmimCl	-391.48	-74.57	-466.05
22	Dimer-C_3mimCl	-387.25	-76.93	-464.18
23	Dimer-BmimCl	-386.93	-75.48	-462.41
24	Dimer-C_4dmimCl	-376.77	-78.23	-455.00

Table S6 H-bond lengths and angles of Dimer-ion pairs conformers at the level of B3LYP/6-31++G** and B3LYP-D3/6-311+G**

	B3LYP/6-31++G**			B3LYP-D3/6-311+G**		
**Structure**	**H-bond**	**Length(Å)**	**Angle (°)**	**H-bond**	**Length(Å)**	**Angle (°)**
Dimer-AmimOAc	C18-H19···O80	2.196	170.75	C18-H19···O80	2.706	105.99
	C3-H8···O81	2.236	164.31	C3-H8···O81	2.345	155.69
	C57-H60···O24	2.212	147.89	C57-H60···O24	2.102	172.56
Dimer-AmimAla	C18-H19···O78	2.212	172.80	C18-H19···O78	2.305	138.38
	C3-H8···O79	2.279	159.91	C3-H8···O79	2.676	102.82
	C57-H60···O24	2.216	147.76	C57-H60···O24	2.244	144.25
Dimer-AmimAsp	C18-H19···O78	2.239	171.09	C18-H19···O78	2.310	136.32
	C3-H8···O79	2.305	159.12	C3-H8···O79	2.663	101.98

	C57-H60···O24	2.202	148.02	C57-H60···O24	2.21	145.64
Dimer-AmimSer	C18-H19···O80	2.213	172.33	C18-H19···O80	2.340	136.52
	C3-H8···O81	2.318	158.11	C3-H8···O81	2.738	102.71
	C57-H60···O24	2.184	150.11	C57-H60···O24	2.167	117.76
Dimer-AmimCl	C15-H16···Cl55	2.610	153.41	C15-H16···Cl55	2.479	159.93
	C26-H29···Cl55	2.853	168.60	C26-H29···Cl55	2.722	178.63
	C58-H61···O13	2.271	146.41	C58-H61···O13	2.248	147.71
Dimer-AmimHSO$_4$	C15-H16···O56	2.315	113.40	C15-H16···O56	2.433	141.14
	C26-H29···O58	2.262	153.98	C26-H29···O58	2.152	153.37
	C63-H66···O13	2.401	127.48	C63-H66···O13	2.162	136.89

Table S7 The main donor-acceptor interactions in the process and their second order perturbation stabilization energy E(2) of Dimer-ion pair.

	**π-stacking interaction, kJ/mol**			**H-bond interaction, kJ/mol**		
**Structure**	**Donor**	**Acceptor**	**E(2)**	**Donor (i)**	**Acceptor (j)**	**E(2)**
Dimer-AmimOAc	πC25-C26	σ*C63-H65	2.89	LP O80	σ* C18-H19	14.83
	πC25-C26	π*C66-C72	0.63	LP O81	σ* C3-H8	9.87
	πC25-C26	π*C63-C65	0.42	LP O24	σ* C57-H60	11.80
Dimer-AmimAla	πC25-C26	σ*C63-H65	2.60	LP O78	σ* C18-H20	14.28
	πC25-C26	π*C66-C72	0.51	LP O79	σ* C3-H8	8.23
	πC25-C26	π*C63-C65	0.39	LPO24	σ* C57-H60	11.59
Dimer-AmimAsp	πC25-C26	σ*C63-H65	3.15	LP O78	σ* C18-H20	12.94
	πC25-C26	π*C66-C72	0.78	LP O79	σ* C3-H8	7.31
	πC25-C26	π*C63-C65	0.37	LPO24	σ* C57-H60	12.47
Dimer-AmimSer	πC25-C26	σ*C63-H65	3.12	LP O78	σ* C18-H19	13.90
	πC25-C26	π*C66-C72	0.23	LP O79	σ* C3-H8	6.72
	πC25-C26	π*C63-C65	0.36	LP O24	σ* C57-H60	13.57
Dimer-AmimCl	πC25-C26	σ*C63-H65	2.56	LP Cl55	σ* C15-H16	10.54
	πC25-C26	π*C66-C72	0.52	LP Cl55	σ* C26-H19	7.06
	πC25-C26	π*C63-C65	0.91	LP O13	σ* C58-H61	8.82
Dimer-AmimHSO$_4$	πC25-C26	σ*C63-H65	2.78	LP O56	σ* C15-H16	11.80
	πC25-C26	π*C66-C72	0.15	LP O58	σ* C26-H29	8.61
	πC25-C26	π*C63-C65	0.68	LP O13	σ* C63-H66	4.2

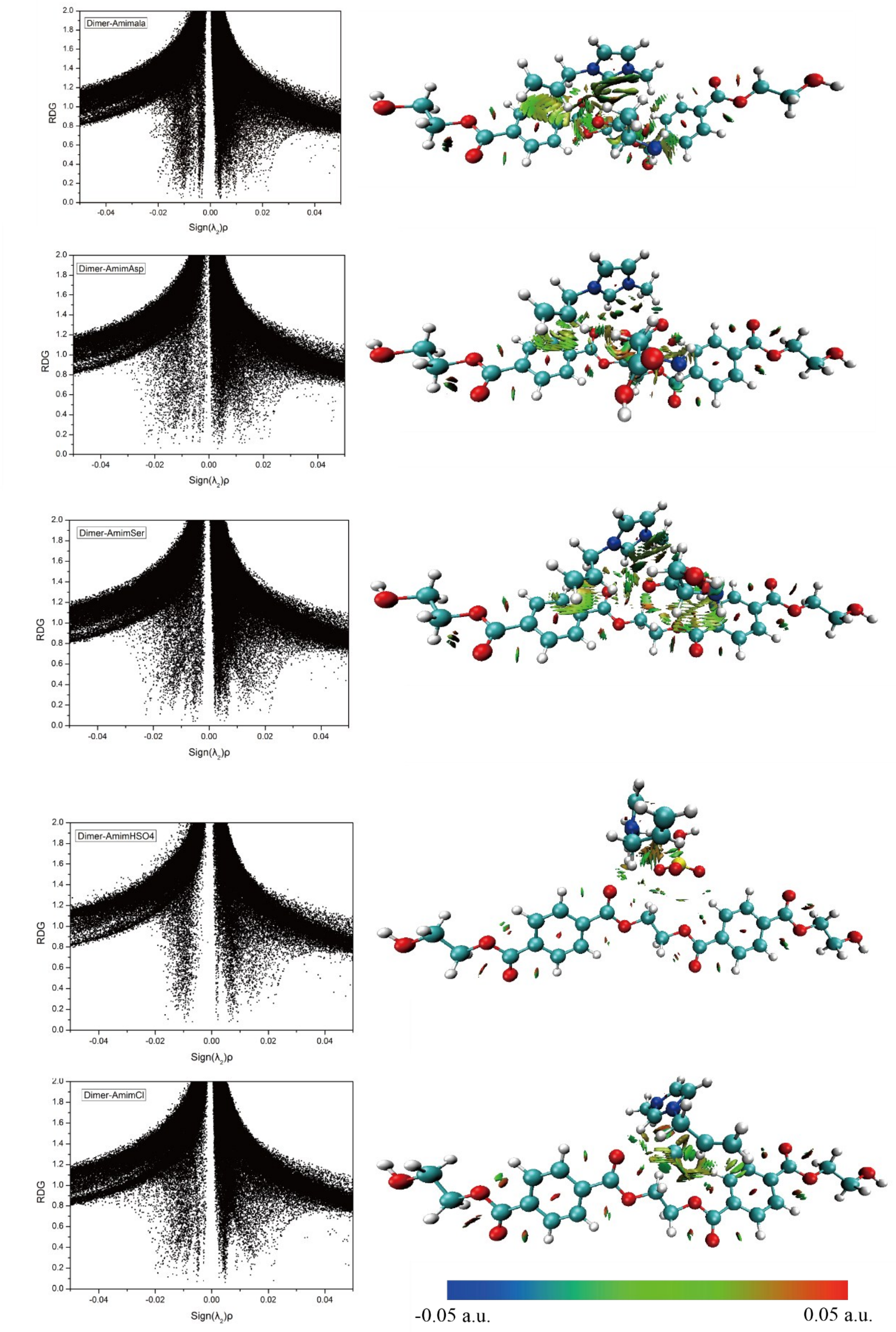

Fig. S9 RDG scatter plot (isovalue =0.5) and isosurfaces (s=0.7) plots of the conformers: Dimer-AmimAla, Dimer-AmimAsp, Dimer-AmimSer, Dimer-AmimCl and Dimer-AmimHSO$_4$. The isosurfaces are colored on a blue-green-red scale according to values of sign(λ_2)ρ, ranging from -0.05 to 0.05 a.u.. Red indicates strong attractive interactions, and blue indicates strong nonbonded overlap.

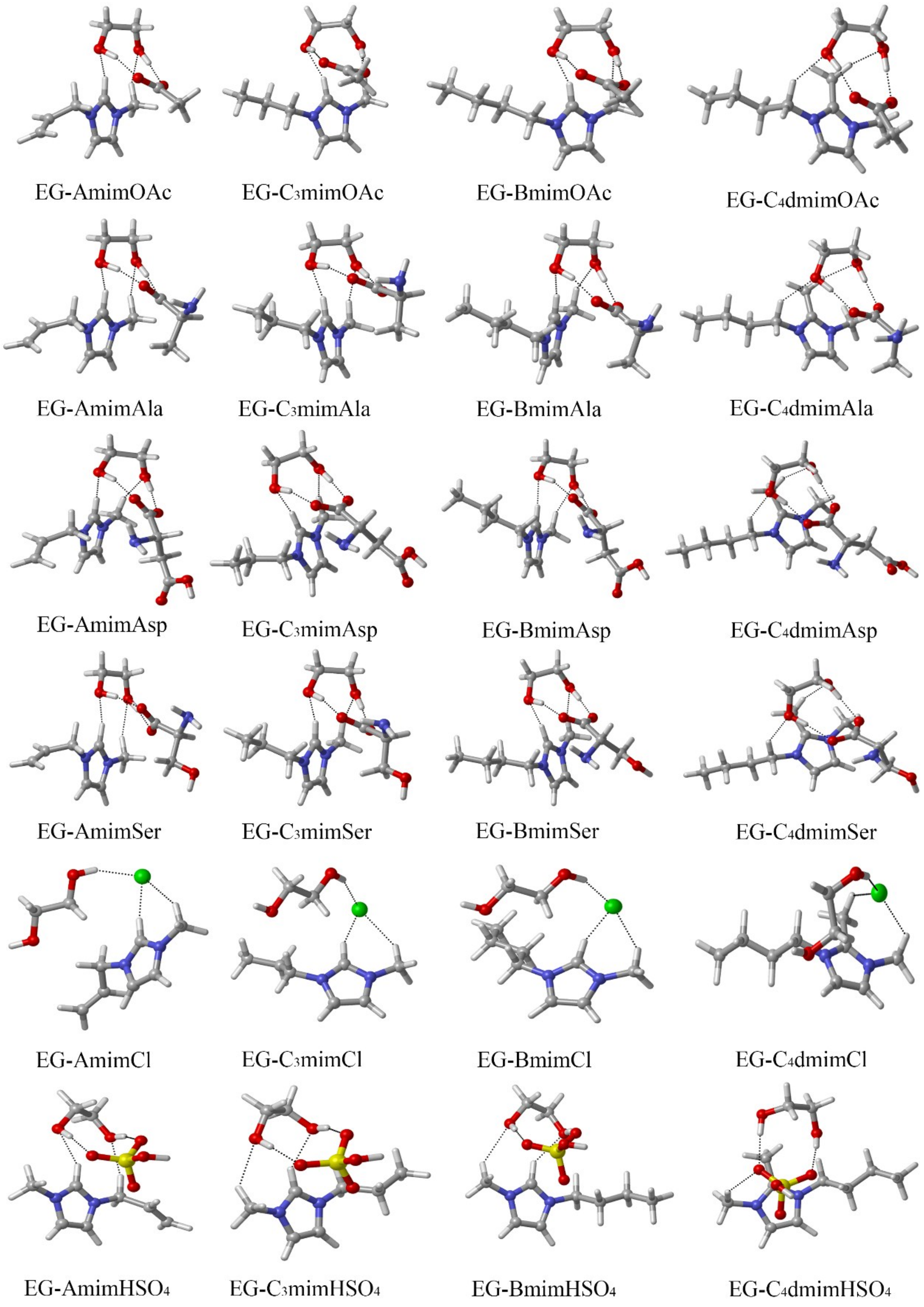

Fig. S10 Results of interaction energy calculation for the 24 kinds of EG and ionic liquids

Table S8 Calculated interaction energy at the B3LYP/6-31++G** level of theory. (E-EG, C-cation, A-anion, CA-ion pairs)

Entry	Structures	ΔE $_{CA}$(kJ/mol)	ΔE$_{E-CA}$ (kJ/mol)	ΔE$_{E-C-A}$ (kJ/mol)
1	EG-AmimOAc	-364.63	-136.86	-501.49
2	EG-C_3mimOAc	-357.35	-141.81	-499.16
3	EG-BmimOAc	-358.07	-139.74	-497.81
4	EG-C_4dmimOAc	-339.11	-133.74	-472.84.
5	EG-AmimAla	-339.64	-142.11	-481.75
6	EG-C_3mimAla	-341.93	-137.29	-479.21
7	EG-BmimAla	-341.94	-135.89	-477.83
8	EG-C_4dmimAla	-320.03	-131.89	-451.92
9	EG-AmimAsp	-329.07	-140.33	-469.40
10	EG-C_3mimAsp	-328.61	-135.21	-463.81
11	EG-BmimAsp	-328.16	-134.30	-462.47
12	EG-C_4dmimAsp	-319.19	-122.65	-441.84
13	EG-AmimSer	-330.82	-141.13	-471.95
14	EG-C_3mimSer	-336.14	-133.63	-469.77
15	EG-BmimSer	-341.44	-138.32	-479.76
16	EG-C_4dmimSer	-315.24	-126.86	-442.10
17	EG-AmimHSO_4	-336.29	-114.00	-450.29
18	EG-C_3mimHSO_4	-331.75	-113.35	-445.09
19	EG-BmimHSO_4	-330.87	-113.02	-443.89
20	EG-C_4dmimHSO_4	-331.46	-88.94	-420.40
21	EG-AmimCl	-374.78	-48.72	-423.50
22	EG-C_3mimCl	-373.97	-47.18	-421.15
23	EG-BmimCl	-372.78	-47.26	-420.04
24	EG-C_4dmimCl	-349.74	-63.43	-413.17

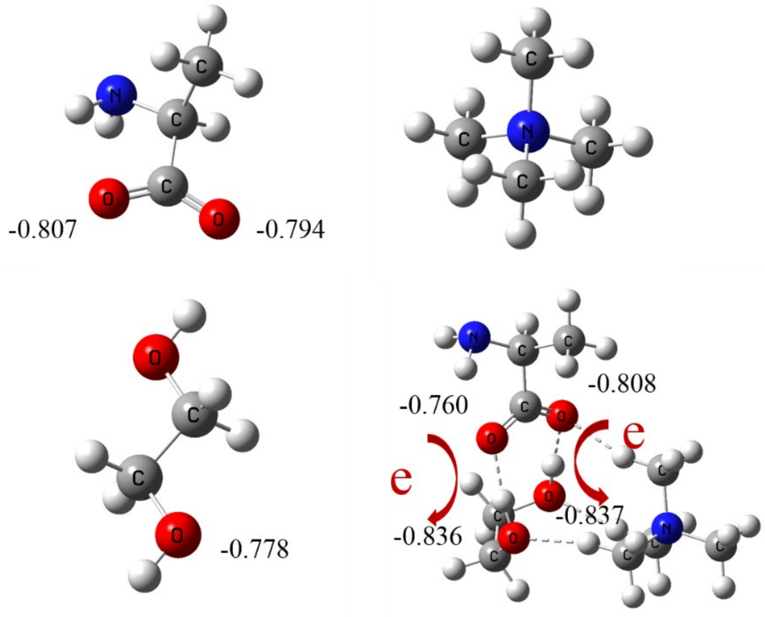

Fig. S11 The charge transfer when anion cation and EG interact

The interaction of ILs and EG makes the electronegativity of oxygen of hydroxyl in EG stronger, which are shown in Fig. S11. The NPA charges of both isolated N_{1111}^{+}, Ala^{-}, EG and EG-N_{1111}Ala are analyzed here. The total negative charge on anion increases from -1 to -0.82. The total positive charge on cation decreases from 1 to 0.90. and the total charge on EG decreases from 0 to -0.08, while a small charge transfer of 0.18 occurs from anion to cation and EG. Fig. S11D shows the direction of the charge transfer. The interaction of anion, cation and EG make the electronegativity of oxygen of hydroxyl in EG stronger than that of before interaction (from -0.778 to -0.836), which make the oxygen in EG prefer to attacking the carbon of the ester group in PET and finally results in degradation of PET.

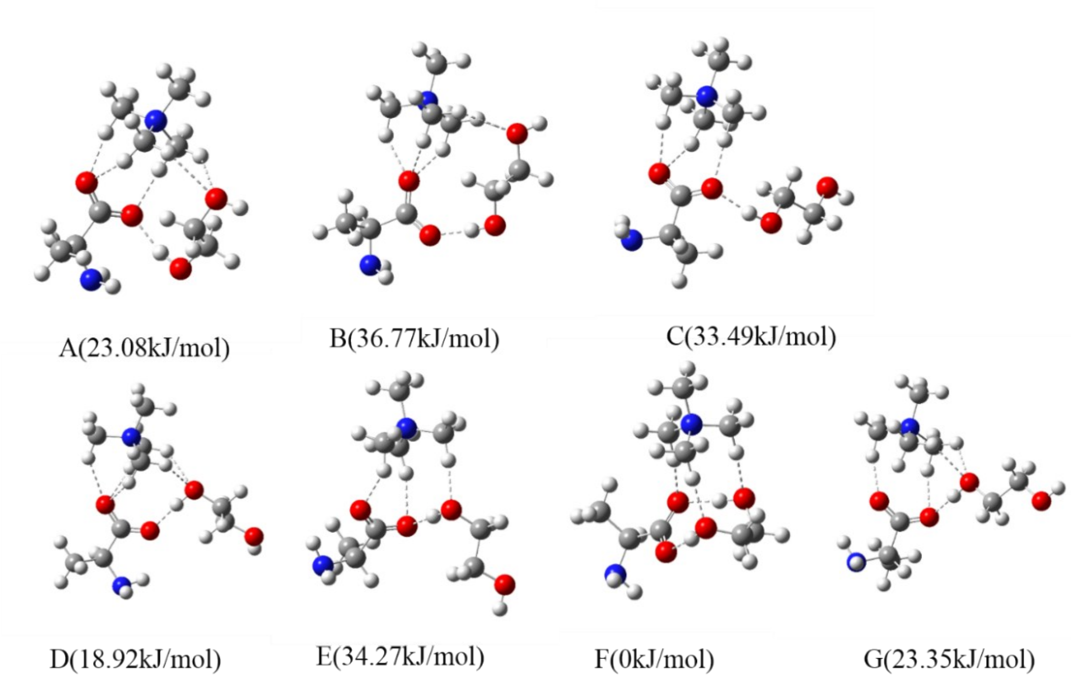

Fig. S12 optimized structures of anion, cation and EG by B3LYP/6-31++G** with the relative energy (kJ/mol). O atoms (red), N atoms (blue), H atoms (light gray), C atoms (dark gray). The dashed lines note the H-bonds formed in the ion pairs

Table S9 Changes in bond length for EG while it interacts with N_{1111}Ala

Bond	EG	A	B	C	D	E	F	G
C1-C4	1.520	1.534	1.528	1.520	1.526	1.526	1.530	1.521
C1-O9	1.429	1.439	1.447	1.405	1.416	1.416	1.417	1.414
C4-O7	1.429	1.406	1.403	1.428	1.425	1.426	1.414	1.422
O9-H10	0.965	0.966	0.964	0.988	1.003	1.004	0.998	0.996
O7-H8	0.965	0.989	0.990	0.965	0.965	0.965	1.002	0.965

The interaction between EG and N_{1111}Ala was studied in Fig. S12. it is found that there are different energy minimum structures (A-G) for the cooperation of EG with N_{1111}Ala via H-bonds. The H-bonds are formed among the oxygen of carboxyl in Ala^-, C-H of N_{1111}^+ and hydrogen of the hydroxyl in EG. Table S8 shows that the interaction between EG and N_{1111}Ala will bring changes to the bond lengths (original 0.965 to 1.004), which renders the length of O-H of EG longer and causes the hydrogen to be lost more easily.
